# Earthquake Experience at Different Trimesters during Pregnancy Is Associated with Leukocyte Telomere Length and Long-term Health in Adulthood

**DOI:** 10.3389/fpsyt.2017.00208

**Published:** 2017-10-16

**Authors:** Ran Wang, Cuixia An, Jincheng Wang, Yumei Wang, Mei Song, Na Li, Yanan Chen, Feifei Sun, Xingshi Chen, Xueyi Wang

**Affiliations:** ^1^Department of Psychiatry, The First Hospital of Hebei Medical University, Shijiazhuang, China; ^2^Mental Health Institute of Hebei Medical University, Shijiazhuang, China; ^3^Brain Ageing and Cognitive Neuroscience Laboratory, The First Hospital of Hebei Medical University, Shijiazhuang, China; ^4^Shanghai Mental Health Center, Shanghai Jiaotong University School of Medicine, Shanghai, China

**Keywords:** leukocyte telomere length, earthquake stress, pregnancy, long-term health, adulthood

## Abstract

Leukocyte telomere length (LTL) is a predictor of age-related diseases, cancer, and even early mortality. Prenatal stress experience has been suggested to associate with short LTL and an increased disease risk in adult life. The present study aimed to evaluate the 39-year effects of prenatal earthquake stress (PES) exposure on LTL and increased age-related disease risk in adulthood. Here, we compared the LTL in the subjects who were exposed to PES to healthy controls (CN) and evaluated whether stress exposure at different times during pregnancy is associated with a shorter LTL and long-term health conditions in adulthood. LTL was measured in 100 adults who experienced the 1976 7.8 Richter scale Tangshan earthquake of the Hebei province *in utero* and divided them into first, second, and third trimester groups according to the exposure timing during pregnancy. A total of 80 healthy volunteers from Shijiazhuang of the Hebei province were also assessed for their LTL. The telomere-to-single copy gene (T/S) ratio of the PES group (0.78 ± 0.06, *p* = 0.04) showed a significantly lower LTL than the CN group (0.97 ± 0.08). The results of the LTL analysis indicated that the subjects who experienced PES in the second (0.69 ± 0.09, *p* = 0.04) or third trimester (0.67 ± 0.76, *p* = 0.02) showed significantly shorter LTLs compared with those in the first trimester group (0.99 ± 0.12). A fully adjusted regression model indicated the same conclusions. In addition, we found that systolic pressure (SBP; 129.32 ± 14.86 mmHg, *p* = 0.041), body mass index (BMI; 22.54 ± 2.71, *p* = 0.046), and low-density lipoprotein (LDL; 3.09 ± 0.98 mmol/L, *p* = 0.048) in the subjects with PES were significantly higher than those measurements in the CN subjects (SBP; 122.06 ± 10.55 mmHg; BMI; 20.24 ± 2.13; LDL; 2.91 ± 0.76 mmol/L), and there was a significant negative correlation between an increased adult hypertension risk and a shorter LTL.

## Introduction

Pregnancy is a complicated and dynamic process. Stressful life events during pregnancy often bring mental stress or trauma to mothers (1–4). Stress can change the flow of blood to the uterus, and the intrauterine environment that is experienced by the fetus, and even induce structural and/or functional alterations in the cell, tissue, and organ system of the fetus, which results in long-term effects in the offspring born from stress-burden mothers (5, 6). Accumulating evidence suggests that the origins of diseases, such as age-related diseases, mental disorders, and cancers, and even various common diseases, could be traced back to the fetal period *in utero* (7–10). Studies also support for an important role of fetal development *in utero* on the health conditions in childhood and adulthood (5, 11). However, the biological mechanisms of these correlations remain unknown. Recent studies support the potential role of telomere/telomerase biology as a mechanism that links offspring who experienced maternal stress during pregnancy and an increased risk of diseases (12–14).

Telomeres act as caps that protect the ends of eukaryotic chromosomes from DNA degradation or repair (15, 16). Telomere shortening occurs in all replicating somatic cells with age, including leukocytes (17). The leukocyte telomere length (LTL) is a biomarker for aging and is associated with age-related diseases (18–21). Some studies indicate that a shorter LTL is linked with high levels of psychosocial stress. Most studies reported that exposure to adverse conditions in infancy or childhood was correlated with a shorter telomere length. Many animal studies have found that early life stress experience is linked to a shorter LTL in the cells of different tissues (22–26). Entringer et al. reported that a shorter LTL is present in newborn and young adult offspring who are exposed to prenatal stress (6, 27). These results suggest that stress-related alterations of biological mechanism can be identified that link psychosocial stress and age-related diseases (28–31). However, no research has investigated an association between the prenatal stress exposure at different trimesters of pregnancy and LTL in adulthood.

An earthquake with a magnitude of 7.8 hit Tangshan, Hebei province, China in the early morning and lasted to 14–16 s and followed by a major 7.1 magnitude aftershock, which resulted in the deaths of 255,000 people and severe injuries of 164,000 people. Such catastrophic earthquake caused extreme stress on pregnant mother and their developing fetuses. In the present study, we hypothesized that the long-term health conditions of offspring who are born from earthquake stress-burden mothers during three different prenatal stages have a shorter LTL in adulthood.

## Materials and Methods

### Samples

A total of 100 subjects whose mothers were exposed to the Tangshan 7.6 Richter scale earthquake were recruited from the workers of the Kailuan Mining group at 6 coal reserve bases, 2 communities, and 2 related units. All of the subjects were divided into three different groups: first trimester (*n* = 32), second trimester (*n* = 34), and third trimester (*n* = 34) groups according to their dates of birth. 80 healthy controls were recruited from the workers of the Shijiazhuang steel mill group, which was 440 km away from Tangshan.

Inclusion criteria for the prenatal stress group being born and raised in Tangshan of the Hebei province, and being born between July 29, 1976 and April 28, 1977.

Inclusion criteria for the control group being born and raised in Shijiazhuang of the Hebei province, and born between July 29, 1976 and April 28, 1977.

Exclusion criteria included epilepsy, hypertension, thyroid disease, diabetes, infection, medication use history, smoking history, other traumatic events (sick, fighting etc.) in addition to earthquake during pregnancy, alcohol use, substance use, psychiatric disorders, medical use, physical illness, and pregnancy. The characteristics of the subjects are shown in Table 1.

**Table 1:** Subject characteristic.

	PES (*n* = 100)	CN (*n* = 80)	*p*-Value
**Current**			
Age (years)	38.59 ± 0.49	39.16 ± 0.95	0.314
Gender (*N*)			
Male	70	64	0.126
Female	30	16	
Education			
High school diploma or higher (*N*)	74	63	0.458
Depressive/anxiety symptom			
HAMD	1.36 ± 0.57	1.14 ± 0.53	0.663
HAMA	3.47 ± 2.92	2.24 ± 1.24	0.375
**History**			
Birth weight	3,173 ± 686.25	3,192.5 ± 518.58	0.108
Length of gestation	39.19 ± 1.22	39.04 ± 1.31	0.376
Natural labor (*N*)	97	76	0.702
Cesarean birth (*N*)	3	4	
CTQ scores			
Emotional abuse	5.23 ± 0.73	5.88 ± 1.18	0.000***
Emotional neglect	9.53 ± 3.95	8.70 ± 3.973	0.688
Sexual abuse	5.073 ± 0.75	5.079 ± 0.60	0.684
Physical abuse	5.26 ± 0.79	5.45 ± 0.91	0.019*
Physical neglect	8.34 ± 2.66	7.45 ± 2.25	0.075
Total CTQ	34.16 ± 7.49	33.43 ± 6.60	0.288
LES scores	20.77 ± 22.73	22.41 ± 22.23	0.433
Disease history during pregnancy	None	None	–
**Factor controlled for study**			
Current chronic diseases	None	None	–
Psychiatry disease/symptom	None	None	–
Smoking	None	None	–
Alcohol	None	None	–
Medication use	None	None	–

*PES, prenatal earthquake stress group; CN, control group; HAMA, Hamilton Anxiety Scale; HAMD, Hamilton Depression Scale; CTQ, Childhood Trauma Questionnaire; LES, Life Event Scale*.

*p < 0.05; ***p < 0.000.

The study received approval from the Ethics Committee of the First Hospital of Hebei Medical University (No. 2014005) and written informed consent in accordance with the Declaration of Helsinki was obtained from all of the subjects before enrollment.

### Questionnaire, and Psychological Evaluation and Assessment

We used questionnaires to collect the earthquake-related pregnancy information. The information was obtained from the parents of subjects or other with knowledge of the event. The traumatic events were evaluated using the Childhood Trauma Questionnaire (CTQ) and the Life Event Scale. The current symptoms of depression/anxiety were assessed using the Hamilton Depressive/Anxiety Scale (HAMD/HAMA) and the Structured Clinical Interview for the DSM-V was used as a diagnostic standard. Psychiatrists performed all of the investigations, and a consistency of 94% was found among all of the investigators.

### Medical Examination

The health condition examination included measurements of height, weight, body mass index (BMI), and blood pressure of prenatal earthquake stress (PES) group. Fasting blood glucose was tested after 8–10 h without food intake. Blood fat levels were measured as triglyceride (TG), total cholesterol, high-density lipoprotein cholesterol, and low-density lipoprotein cholesterol levels.

### Leukocyte Telomere Length

Blood samples were collected in 5 mL EDTA vacuum collection tubes (Inspeck, ST750EK, Sekisui, Osaka, Japan) between 8:00 a.m. and 9:00 a.m. in the morning. The samples were frozen at −80°C until analysis. DNA was extracted from whole blood using a Gentra Puregene Blood Kit (Qiagen, Germantown, MD, USA). DNA was measured for LTL using quantitative polymerase chain reaction as previously published (32) and as previously described (33). The final concentrations of telomere primer were (Tel 1: GGTTTTGAGGGTGAGGGTGAGGGTGAGGGTGAGGGT) 100 nM and (Tel 2: TCCCGACTATCCCTATCCCTTCCCTATCCCTATCCCTA) 300 nM; The final HBG primer concentrations were (HBG1: GCTTCTGACACAACTGTGTTCACTAGC) 200 nM and (HBG2: CACCAACTTCATCCACGTTCACC) 200 nM. The final concentration of reaction mix reagents contained 2× Fast SYBR^®^ Green Master Mix and 10 ng genomic DNA. All PCRs were carried out on a StepOne Plus real-time PCR system (Applied Biosystems, Foster City, CA, USA). The thermal cycling profile consisted of: denature at 95°C for 5 s, anneal at 54°C for 30 s, extend at 72°C for 31 s, 40 cycles. Base pairs (bp) of telomeres in all of the subjects were counted based on the mean fragment length of telomeric restriction from a southern blot analysis, and the slope of the plot of the mean telomeric restriction fragment length vs. the relative T/S ratio for each subject. The conversion formula was bp = 3,274 + 2,413 × (T/S).

### Statistical Analysis

Two groups were compared using an unpaired *t*-test, and a comparison across multiple groups was performed using a one-way ANOVA. The comparison of category data between groups was performed using a chi-square test. Fisher’s least significant difference test was used for *post hoc* analysis.

All of the LTL data were analyzed using a Kolmogorov–Smirnov test (Kolmogorov–Smirnov = 0.976, *p* = 0.538). To assess the association between the fetus born from stress-burden mothers and a shorter LTL in adulthood, a fully adjusted regression model was used that included age, gender and BMI, and potential covariates have been reported in previous studies, such as birth weight, childhood stress exposure, current chronic stress, and depressive/anxiety symptom levels. A standardize effect size (Cohen’s *d* effect size statistic) was used to calculate the difference of the means of two groups. All of the data were analyzed by SPSS 22.0 software and the significant level was *p* < 0.05.

## Results

### Subject Characteristics

Subject characteristics are provided in Table 1. We compared the sociodemographic data, birth outcomes, and some relevant factors that were associated with LTL in previous studies between the prenatal stress group and the healthy control group. Emotional neglect and physical neglect of CTQ in the PES group were significantly lower (emotional neglect, *p* = 0.032; physical neglect, *p* = 0.012) than that in the healthy control group.

### Leukocyte Telomere Length

**Figure 1:**
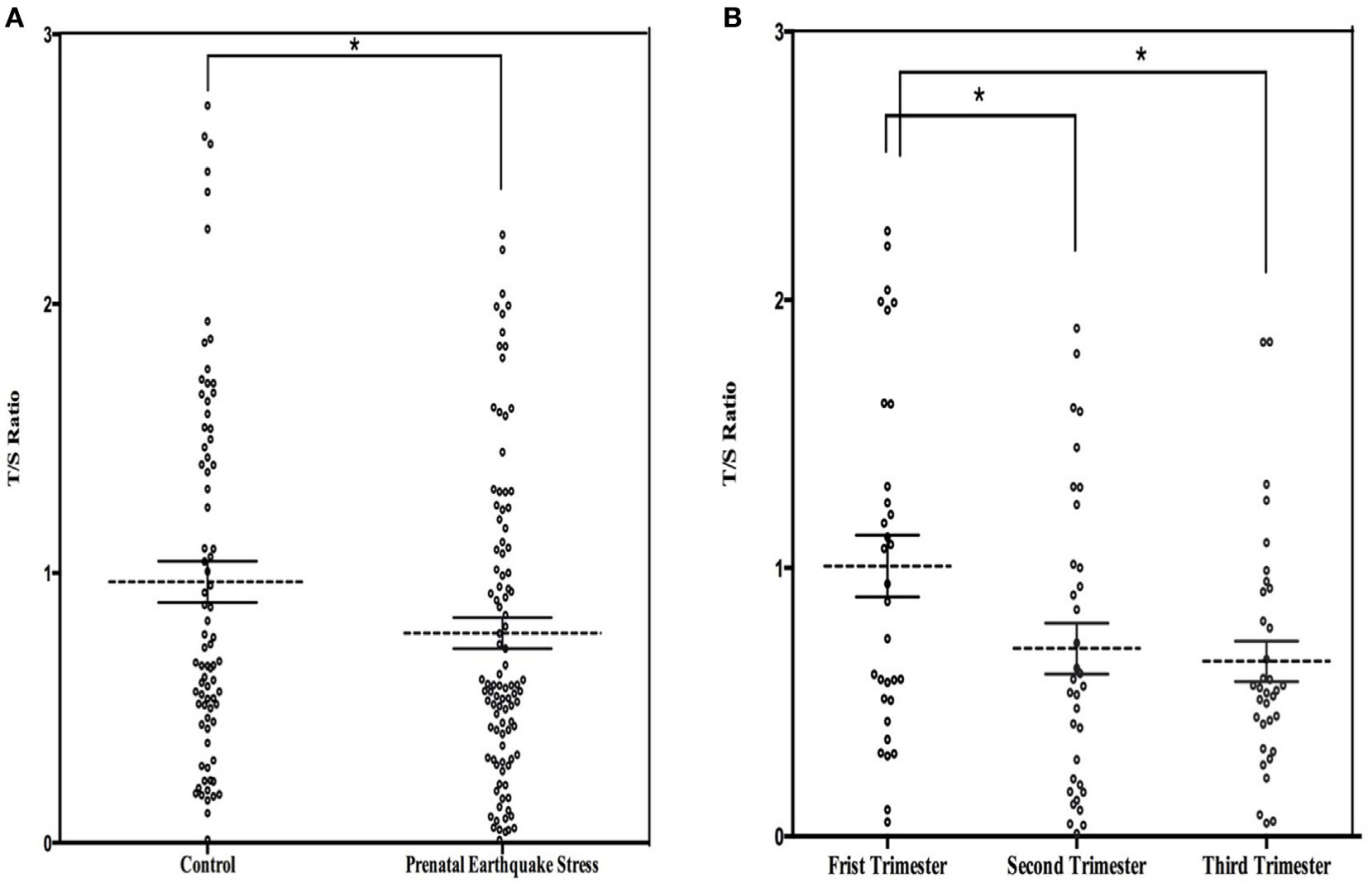
**(A)** Dot plot illustrating leukocyte telomere length (LTL) (T/S ratio) in prenatal earthquake stress (PES) and control subjects; **(B)** dot plot illustrating LTL in subjects who exposed to PES in the three trimesters during pregnancy. The mean levels are marked with horizontal broken lines. The SEs are denoted by the horizontal lines. *p < 0.05.

The mean LTL was a 0.86 ± 0.62 telomere repeat copy number-to-single gene copy number (T/S) ratio (which is equivalent to 5,353 ± 1,516 bp). The data of LTL were normally distributed. A shorter LTL of 460 bp [effect size (Cohen’s *d*) = 0.39 SD units] was found in subjects who experienced PES compared to the control subjects that were not exposed to earthquake stress (Figure 1A). After processing the effects of putative confounding factors (i.e., age, gender, BMI, birth weight, childhood stress exposure, current chronic stress, and depressive/anxiety symptom levels), PES was significantly associated with a shorter LTL (β = −0.070; 95% CI = −0.119 to −0.020; *p* = 0.03) (Table 2). To further assess the effect of earthquake stress on LTL in adulthood, the PES subjects were divided into three subgroups, namely, first, second, and third trimester groups according to their gestational age when earthquake occurred. The result of a one-way ANOVA and *post hoc* analysis revealed that the T/S ratio in the group that experience PES during the second (0.69 ± 0.09; *p* = 0.04 for first vs. second) or third (0.67 ± 0.76; *p* = 0.02 for first vs. third) trimester was significantly lower than those that experienced earthquake stress during the first trimester (0.99 ± 0.12) (Figure 1B).

**Table 2:** Fully adjusted regression predicting leukocyte telomere length (T/S ratio).

	Unstandardized β (95% CI)		
	Whole	Male	Female
Age	−0.036 (−0.071 to −0.002)	−0.047 (−0.091 to −0.003)	−0.074 (0.011 to 0.137)
Gender	−0.051 (−0.074 to −0.012)	–	–
Body mass index	0.013 (−0.029 to 0.055)	0.025 (−0.033 to 0.082)	−0.047 (−0.039 to 0.134)
Prenatal earthquake stress	−0.070** (−0.119 to −0.020)	−0.096** (−0.164 to −0.028)	−0.046* (−0.037 to −0.005)
Birth weight	−0.002 (−0.050 to 0.047)	−0.001 (−0.064 to 0.062)	−0.077 (−0.149 to 0.077)
Emotional abuse	0.016 (−0.033 to 0.064)	0.020 (−0.047 to 0.086)	0.013 (−0.019 to 0.083)
Emotional neglect	0.025 (−0.025 to 0.074)	0.026 (−0.039 to 0.090)	0.056 (−0.062 to 0.049)
Sexual abuse	−0.067 (−0.131 to −0.002)	−0.084 (−0.166 to −0.002)	−0.081 (−0.045 to −0.014)
Physical abuse	−0.048 (−0.094 to −0.002)	−0.007 (−0.096 to 0.082)	−0.033 (−0.083 to 0.107)
Physical neglect	−0.008 (−0.078 to 0.062)	−0.059 (−0.123 to 0.005)	0.066 (−0.040 to 0.007)
LES scores	0.006 (−0.039 to 0.052)	0.005 (−0.056 to 0.067)	−0.004 (−0.049 to 0.082)
HAMA	−0.006 (−0.127 to 0.115)	0.032 (−0.183 to 0.246)	0.012 (−0.141 to 0.313)
HAMD	0.062 (−0.025 to 0.148)	0.094 (−0.039 to 0.227)	−0.180 (−0.063 to 0.101)

*HAMA, Hamilton Anxiety Scale; HAMD, Hamilton Depression Scale; LES, Life Event Scale*.

*p < 0.05; **p < 0.01.

### Comparison of the Health of the PES Group and the Control Group

We also collected medical examination data and found a significant difference between PES and control groups (Table 3). The systolic pressure was significantly higher in the PES group (129.32 ± 14.86 mmHg; *p* = 0.041) compared to that in the control group (122.06 ± 10.55 mmHg). The mean BMI and low-density lipoprotein (LDL) for the PES group was higher (BMI; 22.54 ± 2.71 mmol/L, *p* = 0.046; LDL; 3.09 ± 0.98 mmol/L, *p* = 0.048) compared to the control group (BMI; 20.24 ± 2.13 mmol/L; LDL; 2.91 ± 0.76 mmol/L). No significant differences were found for diastolic pressure, blood sugar, TC, TG, and high-density lipoprotein.

**Table 3:** Health condition.

	PES (*n* = 100)	CN (*n* = 80)	*p*-Value
Systolic pressure (mmHg)	129.32 ± 14.86	122.06 ± 10.55	0.041*
Diastolic pressure (mmHg)	82.97 ± 8.06	82.81 ± 12.03	0.230
Body mass index	22.54 ± 2.71	20.24 ± 2.13	0.046*
Blood sugar (mmol/L)	5.22 ± 0.91	5.19 ± 1.40	0.340
Total cholesterol (mmol/L)	5.19 ± 0.97	4.77 ± 0.96	0.786
Triglyceride (mmol/L)	1.96 ± 0.32	1.83 ± 0.41	0.389
High-density lipoprotein (mmol/L)	1.68 ± 0.38	1.48 ± 0.40	0.591
Low-density lipoprotein (mmol/L)	3.09 ± 0.98	2.91 ± 0.76	0.048*

*PES, prenatal earthquake stress group; CN, control group*.

*p < 0.05.

### Correlation between LTL and Health in the PES Subjects

To estimate the influence of LTL on health in adults who were born from stress-burden mothers during pregnancy, we performed a Pearson correlation analysis (Table 4). There was a markedly negative correlation of systolic pressure (*r* = −0.303; *p* = 0.001) and LTL.

**Table 4:** Correlation between leukocyte telomere length and health condition in prenatal earthquake stress group.

Pearson correlation	*r*	*p*
Systolic pressure (mmHg)	−0.303**	0.001**
Diastolic pressure (mmHg)	−0.048	0.553
Body mass index	−0.102	0.066
Blood sugar (mmol/L)	−0.058	0.449
Total cholesterol (mmol/L)	−0.022	0.783
Triglyceride (mmol/L)	0.043	0.589
High-density lipoprotein (mmol/L)	0.035	0.660
Low-density lipoprotein (mmol/L)	−0.087	0.280

**p < 0.01.

## Discussion

To assess the association between PES and a shorter LTL in adulthood, we measured the LTL of subjects who were exposed to an earthquake during the fetal period and found a significantly shorter LTL compared to subjects who were not exposed to an earthquake during the fetal period. After analyzing the results according to the gestational ages when the earthquake occurred, we found that the LTL of the subjects whose mothers were in the second or third trimester of pregnancy were significantly shorter than the LTL of the subjects whose mothers were in the first trimester. The results did not change after controlling for age, sex, BMI, birth weight, early life events, current chronic stress, and depressive/anxiety symptoms. These findings suggest that shorter LTL in adulthood is associated with the experience of PES, and this association is strongest in the second or third trimester group. Our findings were similar to another related study on an association in PES and control groups (6), but our study is the first to report in humans that exposure to earthquake stress in different trimesters during pregnancy is associated with a shorter LTL. “Biological embedding” is commonly proposed as possible potential mechanism to explain how fetal development adversely affect health, such as prenatal stress and developmental programming leading to adult cardiometabolic disease (34, 35), and telomere length may be the basis for this association (12, 36, 37). A study suggested that a shorter LTL in adulthood largely contributes to crucial factors that exert their influence during the fetal period (38). Our results were consistent with those findings.

Previous studies have indicated that prenatal stress affects the telomere/telomerase biology of the fetus *via* various biological pathways. For example, maternal stress exposure during pregnancy induces an increase of maternal and/or placental hormones, oxidative stress-related mediators, and even inflammatory mediators, which can enter placental–fetal circulation and induce alterations in placental and fetal physiology, including metabolism changes (39, 40). These alterations may affect LTL *via* the regulation the epigenetic status that regulates gene expression across various cells and tissues. In the present study, we found that the individuals who experienced the earthquake in the mid-to-late or late period of pregnancy had significantly shorter LTLs than those in the early period of pregnancy. A study of fetal–placental stress physiology found that placental corticotropin-releasing hormone levels increased by 25% during pregnancy, and the fetus exhibited a rapid growth at 19 weeks in gestation in the mothers who were exposed to trauma. Ellman et al. found that higher levels of maternal cortisol in mothers who were exposed to stress after 31 weeks of pregnancy was associated with a more advanced physical development of the fetus compared to the development 15, 19, and 25 weeks of gestation exposure (41). We hypothesized that maternal stress in the second or third trimester of pregnancy may lead to the release of more stress-related hormones that affect the cells and tissues of the fetus *via* the fetal–placental system compared to that in the first trimester.

Many studies have indicated that obesity, hypertension, and high blood sugar are three physiological conditions that could be elevated by stress to increase the risk of cardiovascular diseases (42–45). Another result in our study indicated that systolic pressure, BMI, and blood sugar, which are cardiovascular risk-related factors, in the offspring born from stress-burdened mothers during pregnancy were significantly higher than the control group. This finding suggested that PES is linked with an increased disease risk in adulthood. Telomeric DNA is progressively lost with each cell division, but critically telomere shorten occurs during exposure to somatic and/or psychological stress. Greater release of inflammatory factors can enter to the fetus by placental–fetal circulation during maternal stress exposure, which is associated with inflammation-oxidative stress pathway, would induce an increased cell division of fetus and result in a higher risk of cardiovascular diseases in adulthood. Meanwhile, increased cell division would accelerate telomeres shorten in cells that continued to divide in the periphery (46, 47). In present study, Pearson correlations suggested that a shorter LTL in adulthood was correlated with an increased hypertension disease risk in subjects who experienced PES. Our findings suggested that LTL would be a potential biomarker of prenatal stress-related cardiovascular diseases.

We also evaluated the potential confounding factors, such as age, sex, BMI, early life events, childhood trauma, and current stress conditions (symptoms of depression or anxiety). The influence of PES was unchanged after adjusting for these factors. Many studies have reported that sex differences in early life stress exposures. Unfortunately, we could not examine the possibility of significant sex-specific programming effects because the occupational characteristics of the subjects in this study caused a larger number of differences.

Although our study found support that telomere biology is a potential mechanism that links LTL and prenatal stress exposure, future studies must be conducted with a larger sample to examine sex-specific effects. Another limitation of our study is that we used Shijiazhuang as a control group, which in the same province with Tangshan, more work still need to be done examining other confounding factors (e.g., family condition, diet, parenting, and environmental factors) to improve current results. Also additional studies should use animal models to investigate the specific biological mechanism of the prenatal stress-induced increase of the risk of age-related disease.

## Ethics Statement

The study received approval from the Ethics Committee of the First Hospital of Hebei Medical University and written informed consent in accordance with the Declaration of Helsinki was obtained from all of the subjects before enrollment.

## Author Contributions

XW, XC, and RW designed the paper. RW, NL, YC, and FS performed the experiment. CA, YW, and MS analyzed the data. RW wrote the manuscript. All authors read and commented on the manuscript.

## Conflict of Interest Statement

The authors declare that the research was conducted in the absence of any commercial or financial relationships that could be construed as a potential conflict of interest.
